# Cellular and synaptic specializations for sub-millisecond precision in the mammalian auditory brainstem

**DOI:** 10.3389/fncel.2025.1568506

**Published:** 2025-05-19

**Authors:** Christian Keine, Bernhard Englitz

**Affiliations:** ^1^Carl von Ossietzky Universität Oldenburg, School of Medicine and Health Sciences, Division of Physiology, Oldenburg, Germany; ^2^Research Center Neurosensory Science, Carl von Ossietzky Universität, Oldenburg, Germany; ^3^Computational Neuroscience Lab, Donders Center for Neuroscience, Radboud University, Nijmegen, Netherlands

**Keywords:** synaptic transmission, auditory brainstem, specialization, cochlear nucleus (CN), medial nucleus of the trapezoid body (MNTB), ventral nucleus of the lateral lemniscus (VNLL), medial superior olive (MSO), lateral superior olive (LSO)

## Abstract

Audition in all animals relies on delicate sound pressure variations arriving at the ears, and these sound waves are intertwined representations of the complex auditory environment. The process of auditory perception and behavior is fundamentally based on reconstructive analysis, starting at the auditory nerve and culminating in the segregation of auditory sources through the extraction of spatial, spectral, and temporal cues. This analysis is made possible by specialized structures in the auditory brainstem that accurately represent and process incoming signals, preparing them for various downstream analyses. Decades of research have provided substantial insight into the morphological and physiological adaptations of specific auditory synapses, which we present and compare in the context of their presumed functions. Here, we focus on two parallel pathways originating from the auditory nerve and converging in the midbrain, featuring several well-studied synapses across multiple nuclei (cochlear nucleus, medial nucleus of the trapezoid body, ventral nucleus of the lateral lemniscus, and medial and lateral superior olivary nuclei). These synapses form the backbone of the high temporal precision of auditory representation, which is crucial for sound localization, speech comprehension, and speaker identification, each relying on subtle monaural or binaural cues. Finally, we highlight the similarities and differences with other brain areas that face challenges comparable to those of the auditory system.

## Introduction

The auditory system has the remarkable ability to effortlessly decode the complex mixture of sounds around us, locating a person's voice in a noisy room, appreciating the nuances of music, or reacting instantly to warning sounds. This ability hinges on the capacity to process information with extraordinary speed and precision, resolving details on a sub-millisecond timescale. Here, the principle of “form follows function” is illustrated at multiple levels across different species and brain structures. The neuronal processing architecture and cellular components of the system have been shaped by the specific requirements for speed, temporal precision, and faithful reconstruction. This process starts in the cochlea, where low-dimensional, high-sampling-rate sound pressure fluctuations are converted into a high-dimensional, lower-sampling-rate representation of the electrical signals. The electrical information is then transmitted via the auditory nerve to the brainstem, where it is processed and relayed through various ascending pathways, beginning with the cochlear nucleus. Complex computations essential for sound localization and understanding intricate patterns, such as speech, must occur at these early stations, as microsecond accuracy cannot be maintained for long in a complex, constantly varying system, such as the brain. Maintaining a tight temporal relationship between spike times and the temporal features of sound is required to address these challenges. To meet these demands, several parallel pathways have evolved to extract specific sound features and serve complementary roles in sound processing, each with intriguing specializations. Failure to develop or maintain these extraordinary properties may lead to deficiencies such as auditory processing disorders, delayed language development in children, and deficiencies in sound localization, binaural hearing, speech perception in noise, and tinnitus (Whitton et al., 2011; Gourévitch et al., 2014; Kopp-Scheinpflug and Tempel, 2015; Kaplan et al., 2016; Shore and Wu, 2019; Jacxsens et al., 2022; Knipper et al., 2022).

In this review, we focus on the cellular and synaptic specializations of two parallel pathways: the binaural localization pathway starting from the bushy cells of the cochlear nucleus (CN), including the medial nucleus of the trapezoid body (MNTB), and the two primary binaural computation centers in the superior olivary complex (SOC), the medial and lateral superior olivary nuclei (MSO and LSO). Second, we highlight the specializations in a monaural “spectral feature extraction pathway,” starting from the octopus cells in the CN and including globular cells in the ventral nucleus of the lateral lemniscus (VNLL). Both pathways start by receiving input from the auditory nerve and constitute major inputs to the inferior colliculus (IC), a midbrain integration center. We aimed to examine the current understanding of specializations at various levels, including morphological and physiological adaptations, and interpret them in light of their neuronal circuit function. We conclude this review by discussing the functional implications and significance of comparable specializations in other sensory systems.

## Anatomical overview and present focus

**Figure 1:**
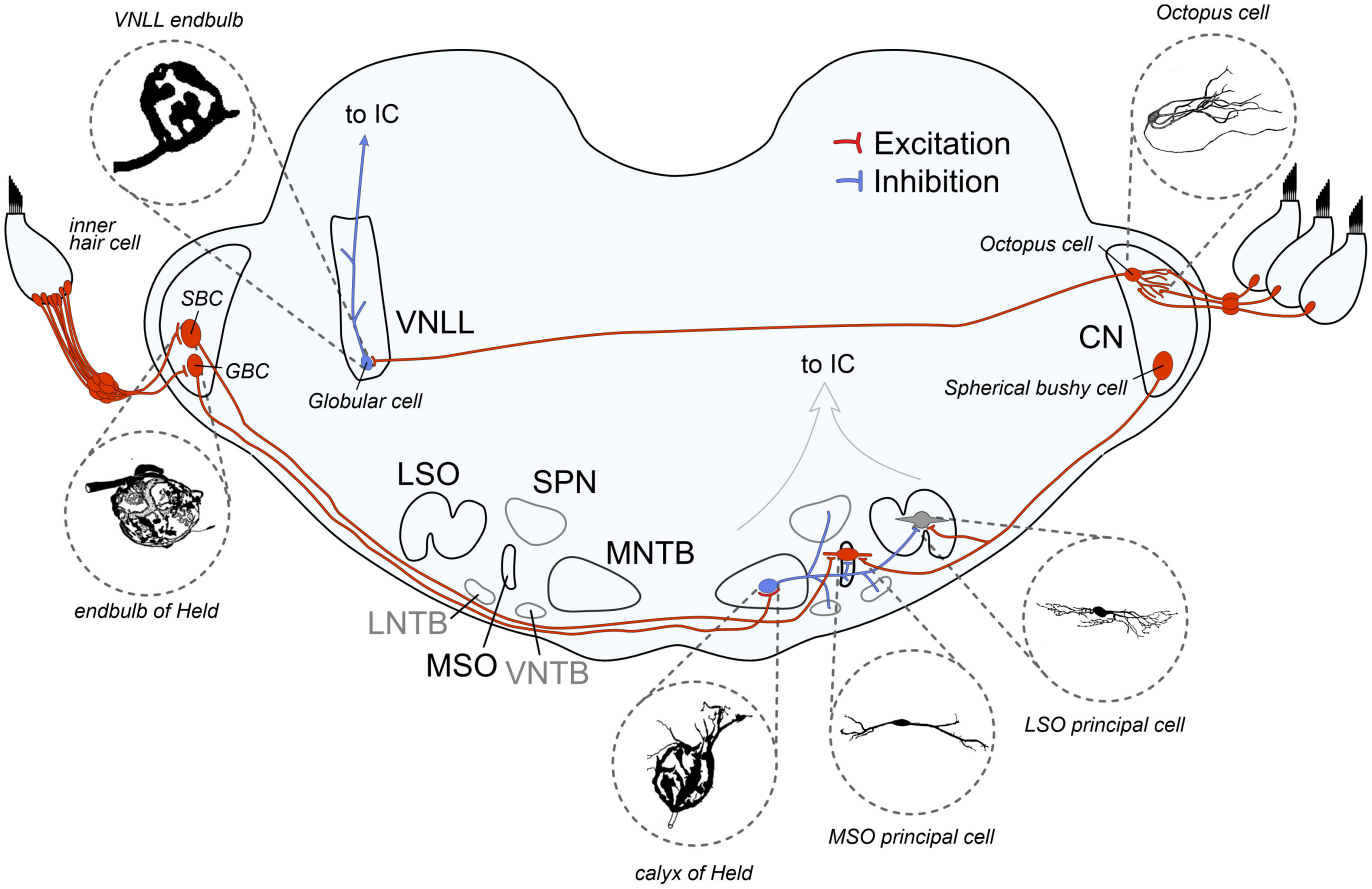
Schematic overview of auditory brainstem pathways discussed in this review. The binaural pathway includes two giant axosomatic terminals in the cochlear nucleus (CN) and the medial nucleus of the trapezoid body (MNTB) before making contact onto the binaural coincidence detector neurons that calculate interaural time and level differences in the medial and lateral superior olive (MSO and LSO, respectively). The monaural pathway to detect spectral sound features includes the octopus cells in the CN as monaural coincidence detectors and the giant axosomatic endbulb onto globular cells in the ventral nucleus of the lateral lemniscus (VNLL). LNTB, lateral nucleus of the trapezoid body; VNTB, ventral nucleus of the trapezoid body; SPN, superior olivary nucleus; IC, inferior colliculus; SBC, spherical bushy cell; GBC, globular bushy cell. Image sources: endbulb of Held: modified from Ryugo et al. (1998) with permission (John Wiley and Sons), calyx of Held: modified from Morest (1968) with permission (Copyright 1968 Springer-Verlag), VNLL endbulb: modified from Vater and Feng (1990) with permission (John Wiley and Sons), MSO principal cell: modified from Scott et al. (2005) (Copyright 2005 Society for Neuroscience), LSO principal cell: modified from Rietzel and Friauf (1998) with permission (John Wiley and Sons), Octopus cell: modified from Oertel et al. (2000) (Copyright 2000 National Academy of Sciences).

Sound processing begins in the cochlea, where sound frequencies are spatially mapped along the basilar membrane. This low-dimensional representation of sound information at the sensory epithelium required the evolution of separate specialized pathways to reconstruct and analyze different aspects of the auditory environment, resulting in parallel streams of information. Frequency-specific information from the cochlea is relayed to the central auditory system via spiral ganglion neurons (SGNs), whose axons collectively form the auditory nerve (AN; Figure 1). These thick, myelinated axons are excitatory (glutamatergic) and make contact with various cell types in the CN (Ryugo and Fekete, 1982; Gómez-Nieto and Rubio, 2011), the starting point of multiple parallel processing pathways, each extracting different features of the auditory scene.

**Figure 2:**
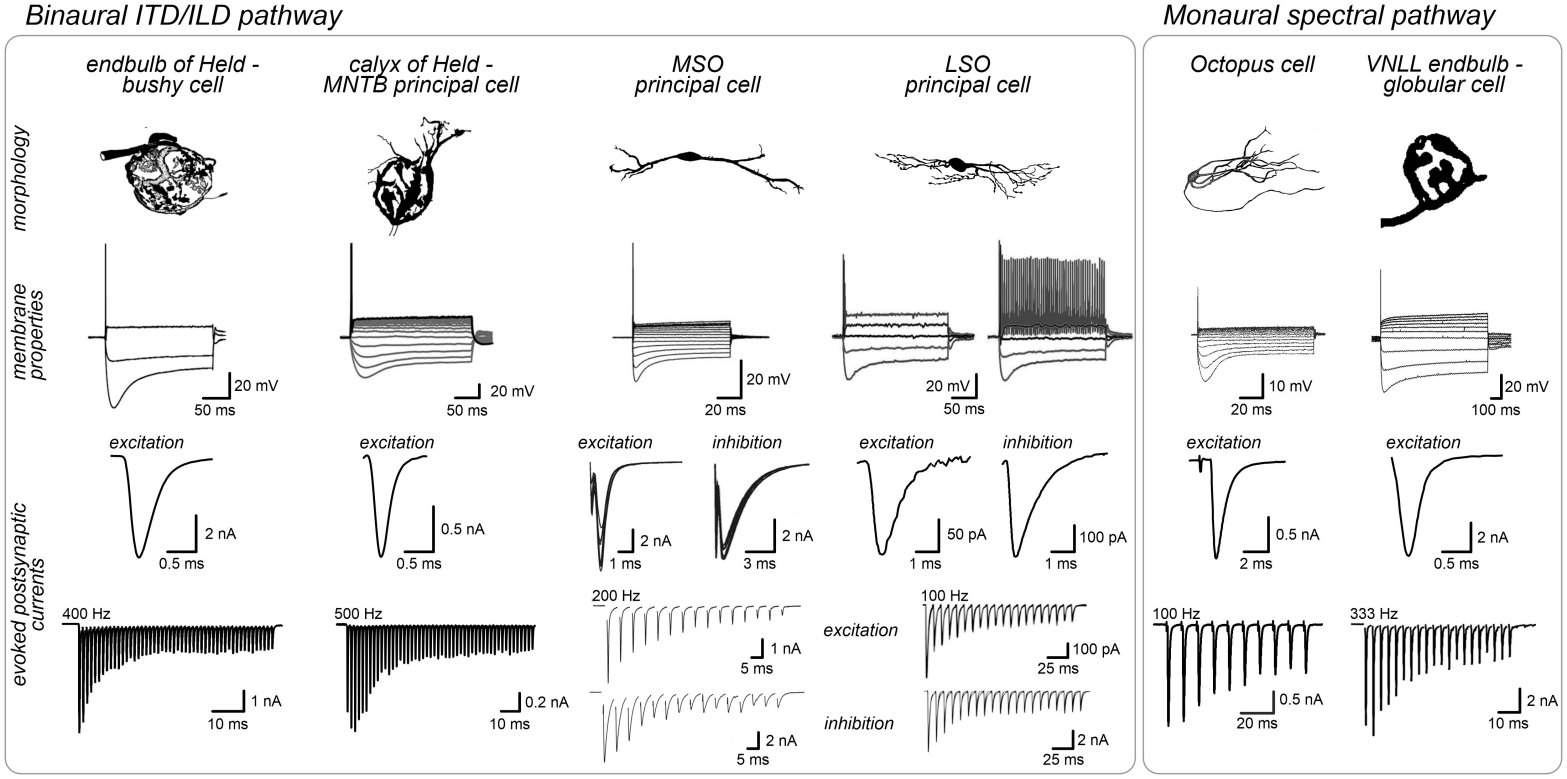
Shared structural and physiological adaptations facilitate rapid signal processing across auditory brainstem pathways. Neurons specialized for high-fidelity synaptic transmission such as bushy cells, MNTB neurons and VNLL globular cells often feature large round cell bodies targeted by giant axosomatic terminals (endbulbs/calyces) that evoked strong and rapid synaptic currents. These cells typically have moderate input resistance. In contrast, neurons acting as coincidence detectors, like MSO and LSO principal neurons (binaural pathway) or octopus cells (monaural pathway) tend to have more complex cell morphologies, integrate multiple smaller inputs along their dendrites and exhibit exceptionally fast membrane properties (low input resistance, fast time constant) ideally suited for precise temporal integration of fast synaptic currents. Post-synaptic current amplitudes are illustrative and depend on experimental conditions, thus limiting a direct comparison between studies. Image sources: endbulb of Held: morphology modified from Ryugo et al. (1998) with permission (John Wiley and Sons), membrane properties modified from Nerlich et al. (2014b), synaptic currents modified from Xie and Manis (2017); calyx of Held: morphology modified from Morest (1968) with permission (Copyright 1968 Springer-Verlag), membrane properties modified from Weimann et al. (2024), synaptic currents modified from Keine et al. (2022); MSO, morphology and membrane properties modified from Scott et al. (2005) (Copyright 2005 Society for Neuroscience), synaptic currents modified from Couchman et al. (2010); LSO, morphology modified from Rietzel and Friauf (1998) with permission (John Wiley and Sons), membrane properties modified from Haragopal and Winters (2023) (CC BY 4.0), synaptic currents modified from Pilati et al. (2016) and Garcia-Pino et al. (2017). Octopus cell: modified from Oertel et al. (2000) (Copyright 2000 National Academy of Sciences), membrane properties: modified from Golding et al. (1999) (Copyright 1999 Society for Neuroscience), synaptic currents modified from Cao and Oertel (2010) (Copyright 2010 The American Physiological Society); VNLL: morphology modified from Vater and Feng (1990) with permission (John Wiley and Sons), membrane properties modified from Caspari et al. (2015), synaptic currents modified from Kladisios et al. (2022).

The two pathways selected in this review, both starting from the AN and converging in the IC, share the presence of giant axosomatic terminals, alongside more conventional synaptic boutons. The primary binaural pathway begins with the endbulb of the Held terminal onto CN bushy cells and includes the calyx of the Held—MNTB synapse, as well as the two binaural nuclei MSO and LSO for computing interaural time and level differences (ITDs and ILDs, respectively). This information is then used to localize sound sources in the horizontal plane and enables us to turn our eyes, head, and body toward a sound, which is fundamental to identifying threats, finding resources, and focusing attention. In parallel, a distinct monaural pathway specialized for processing spectrotemporal sound features is critical for detecting temporal gaps and onsets, which is critical for speech understanding and listening in noise (Verhey et al., 2003). This circuit includes octopus cells in the CN and endbulb-like synapses on globular cells in the VNLL (see Figure 2 and Table 1 for an overview). Its ability to precisely encode transient events also provides cues for segregating competing sounds in auditory scene analysis.

**Table 1:** Cellular and synaptic properties of auditory brainstem neurons in two parallel pathways.

		**Interaural ITD/ILD pathway**						**Monaural spectral pathway**	
	**Structure**	**Endbulb of Held – bushy cell**	**Calyx of Held - MNTB**	**MSO**		**LSO**		**Octopus cell**	**VNLL endbulb – globular cell**
	**Synapse type**	**Excitatory (glutamate)**	**Excitatory (glutamate)**	**Excitatory (glutamate)**	**Inhibitory glycine)**	**Excitatory (glutamate)**	**Inhibitory (glycine)**	**Excitatory (glutamate)**	**Excitatory (glutamate)**
**Pre-synaptic**	Morphology	Large, cup-like or finger-like, complex	Giant, cup-like or finger-like, complex	Bouton-type with multiple varicosities	Bouton-type with multiple varicosities	Bouton-type with few varicosities	Large bouton-type with multiple varicosities	Bouton-type	Large, cup-like, simple
	Number of inputs	1–4 (SBC) 5–60 (GBC)	1	4–8	2–4	20–40	5–8	>60	1–3
	Active zone number	150–2,000	500–1,000	~5 per bouton	-	1–3 per input	6–60 per input	-	-
	Dominant VGCC subtype	CaV2.1	CaV2.1	CaV2.1/CaV2.2	CaV2.1	CaV2.1	CaV2.1	-	-
**Post-synaptic**	Morphology	Globular with 1–2 tufted dendrites	Globular with tufted dendrites	Bipolar with thick dendrites		Bipolar with intricately branched dendrites		Globular with 4–6 thick undirectional dendrites	Globular with profuse dendrites
	Membrane time constant	< 2 ms	2–4 ms	0.3–0.5 ms		1–3 ms		~0.2 ms	2–4 ms
	Input resistance	~100 MΩ	~100 MΩ	< 10 MΩ		~50 MΩ		< 10 MΩ	100–150 MΩ
	AP half-width	200–500 μs	~200 μs	~300 μs		~200 μs		~300 μs	200–500 μs
	HCN expression	HCN1,2,4	HCN2	HCN1		HCN1,2		HCN1	HCN1,2

For each synapse, we analyze three core aspects that underpin their specialized functions. First, we investigate the general structure and morphology and compare the prevalence of these structures across species. Second, we focus on the organization and structure of synaptic inputs, as their number and location on the post-synaptic cell are critical in shaping the output. Third, we examine pre- and post-synaptic physiology, since regulating neurotransmitter release is essential for synaptic function and, together with the intrinsic properties of post-synaptic cells, provides the basis for information processing throughout the ascending auditory pathway. By comparing these features across different cell types and nuclei, we aim to illustrate the strategies employed by the auditory brainstem to achieve its remarkable processing capabilities.

## The bushy cell—superior olive pathway

Sound localization relies on the computation of interaural differences and depends on the temporal acuity of signal transmission. The bushy cell—superior olive pathway is critical for preprocessing acoustic information and transmitting it to the centers of binaural integration. At the beginning of this pathway, bushy cells in the CN receive excitatory inputs from auditory nerve fibers via giant axosomatic terminals called the endbulbs of Held (Held, 1891; Brawer et al., 1974; Lorente de Nó, 1981; Ramon y Cajal, 1995). Bushy cells are excitatory (glutamatergic) and project to nuclei in the SOC including the MNTB, LSO, and MSO (Cant and Casseday, 1986). The MNTB features a second giant axosomatic terminal, the calyx of Held, which is perhaps the largest and best-studied synapse in the central nervous system. The two binaural nuclei, the MSO and LSO, compute ITDs and ILDs, respectively. Despite differences in axonal length and an additional chemical synapse in the MNTB, this pathway operates with astonishing accuracy and allows the detection of ILDs of <1 dB and ITDs as small as 10 μs, which is approximately 100 times faster than the average duration of an action potential (Klumpp and Eady, 1956; Mills, 1958; Thavam and Dietz, 2019). Achieving this precision requires exceptional timing across several synapses, which has been achieved through cellular and synaptic specializations in this pathway.

### The endbulb of Held

#### Structure and morphology

The first central synapse in this pathway is established between the auditory nerve (AN) and bushy cells in the CN. This specialized connection, known as the endbulb of Held, is conserved across the animal kingdom, including reptiles (Browner and Marbey, 1988; Szpir et al., 1990), birds (Carr and Boudreau, 1991; Köppl, 1994), and mammals such as mice (Limb and Ryugo, 2000), cats (Sento and Ryugo, 1989; Ryugo et al., 1998), dolphins (Malkemper et al., 2012), primates (Gómez-Nieto and Rubio, 2011), and humans (Adams, 1986) (see Ryugo and Parks, 2003 for review). Bushy cells are large, round cells characterized by one, or occasionally two, short, bushy dendritic trees (Brawer et al., 1974). In species with extended low-frequency hearing, such as cats and gerbils, bushy cells are classified into two types based on the distribution of endoplasmic reticulum when viewed under an electron microscope: spherical (SBC) and globular bushy cells (GBC) (Cant and Morest, 1979b; Tolbert and Morest, 1982). Although this morphological distinction is less evident in species like mice with limited low-frequency hearing (Lauer et al., 2013), a recent study confirmed the presence of distinct bushy cell types based on transcriptome analysis (Jing et al., 2025). In cats, SBCs are further subdivided into small and large cells based on their soma sizes, with distinct projection patterns to the LSO and MSO, respectively (Osen, 1969b; Cant and Casseday, 1986). This classification into small and large SBCs also appears in humans (Wagoner and Kulesza, 2009) but has not been observed in gerbils (Gleich et al., 1998; Bazwinsky et al., 2008) or guinea pigs (Hackney et al., 1990). SBCs and GBCs occupy different regions in the CN: SBCs are located rostrally in the anteroventral CN (AVCN), with the largest cells occupying the most rostral and ventral portion, where they respond to low-frequency sounds (Osen, 1969b; Brawer et al., 1974; Sento and Ryugo, 1989; Liberman, 1991). GBCs are clustered around the auditory nerve root, the caudal AVCN, and the posteroventral CN (PVCN) (Osen, 1969a; Cant and Morest, 1979a; Tolbert et al., 1982; Wagoner and Kulesza, 2009).

Despite being part of the same neuronal circuit, SBCs and GBCs have distinct projection targets. SBCs project to the MSO (bilaterally) and the ipsilateral LSO (Osen, 1969b; Cant and Casseday, 1986). In contrast, GBCs primarily target the contralateral MNTB, via the large calyx of Held, while also sending projections to the ipsilateral LSO (Harrison and Warr, 1962; Tolbert et al., 1982; Smith et al., 1991).

#### Organization of synaptic inputs

The number, strength, and distribution of synaptic inputs on the post-synaptic cell are critical for shaping the post-synaptic output. Specifically, the probability of spike generation depends on these properties and generally increases with the number, strength, and proximity of synaptic inputs to each other and the soma.

The number of endbulb inputs on bushy cells can vary significantly depending on the cell type, species, and location within the CN. In cats, the largest SBCs, which are tuned to low frequencies and located in the rostral pole of the AVCN, receive input from only 1–2 large endbulbs (Brawer and Morest, 1975; Rouiller et al., 1986; Sento and Ryugo, 1989; Ryugo and Sento, 1991; Jones et al., 1992), whereas in mice, SBCs are contacted by approximately 2–4 endbulbs (Cao and Oertel, 2010; Wright et al., 2014). In contrast, GBCs receive substantially more endbulb inputs, ranging from 5–12 in mice (Cao and Oertel, 2010; Spirou et al., 2023) to approximately 20 in cats, although some cells can receive up to 60 endbulb inputs (Liberman, 1991; Ostapoff and Morest, 1991; Spirou et al., 2005). Further complexity exists in mice, where multiple endbulbs contacting an individual BC can originate from different SGN subtypes (Wang et al., 2021; Wong et al., 2025) and vary significantly in size and synaptic strength, resulting in 1–2 dominant suprathreshold inputs alongside multiple smaller endbulb inputs (Spirou et al., 2023). However, in cats, individual bushy cells receive inputs with similar morphologies, presumably from the same SGN subtype (Sento and Ryugo, 1989; Ryugo and Sento, 1991). Despite being a large axosomatic synapse, the endbulb's intricate morphology allows individual swellings to form connections with the somata and dendrites of nearby neurons, creating neuronal clusters; however, their physiological role remains unclear (Cant and Morest, 1979b; Smith and Rhode, 1987; Ostapoff and Morest, 1991; Ryugo and Sento, 1991; Ryugo et al., 1996; Spirou et al., 2005; Gómez-Nieto and Rubio, 2009). The endbulb's exceptional size accommodates many individual release sites (active zones, AZ), ranging from 150 in rats (Nicol and Walmsley, 2002) to 500–2,000 in cats (Cant and Morest, 1979b; Ryugo and Fekete, 1982; Ryugo et al., 1996). Despite the large morphological scale of the terminal, the ultrastructure of individual AZs resembles that found in conventional bouton synapses, containing approximately 2–4 “docked” vesicles ready for release (Ryugo et al., 1996; Nicol and Walmsley, 2002; Ryugo et al., 2006; Lin et al., 2011; Mendoza Schulz et al., 2014). Consequently, the endbulb possesses a substantial readily-releasable pool (RRP) of several hundred synaptic vesicles (Nicol and Walmsley, 2002; Lin et al., 2011; Taruno et al., 2012; Butola et al., 2021; Hintze et al., 2021). Another distinguishing feature to conventional synapses is that endbulbs contain a dense network of mitochondria (Lenn and Reese, 1966; Tolbert and Morest, 1982; Lauer et al., 2013; Hintze et al., 2024). The abundance of pre-synaptic mitochondria is thought to meet the high metabolic demands of sustained firing and potentially help buffer calcium within the terminal (Rowland et al., 2000; Billups and Forsythe, 2002; Kim et al., 2005; Verstreken et al., 2005; Perkins et al., 2010; Lucas et al., 2018).

#### Pre- and post-synaptic physiology

Beyond organization and morphology, synaptic function relies on the finely tuned interplay between pre- and post-synaptic terminals, where many adaptations are observed at the physiological level. Ion channels and receptors on both sides ultimately shape the input-output function and determine the computational capacity of the neuronal circuits. As one of the largest pre-synaptic terminals, the endbulb of Held is accessible to whole-cell patch-clamp recordings, allowing measurement of action potential (AP) properties and calcium currents, both of which influence synaptic vesicle release (Lin et al., 2011; Butola et al., 2021). Pre-synaptic APs in the endbulb are brief (half-width: 200–300 μs), and this brevity limits the duration of calcium influx into the pre-synaptic terminal, thus effectively regulating synaptic vesicle (SV) release (Lin et al., 2011).

Calcium enters the pre-synaptic terminal through voltage-gated calcium channels (VGCC), which triggers the release of SV in response to a pre-synaptic AP (Nanou and Catterall, 2018; Young and Veeraraghavan, 2021). In the mature endbulb, the dominant VGCC subtype is Ca_V_2.1 (P/Q-type) (Doughty et al., 1998; Lin et al., 2011; Zhuang et al., 2020), which opens during short APs and triggers efficient SV release (Scheuber et al., 2004; Eggermann et al., 2011; Dolphin and Lee, 2020). Although physiological estimates suggest that the total number of VGCC in the endbulb is lower than that in the calyx (Lin et al., 2011), the VGCC density per AZ is comparable (endbulb: ~40 VGCC/AZ, calyx of Held: ~50 VGCC/AZ) when adjusted for terminal size and AZ number (Lin et al., 2011; Nakamura et al., 2015; Lübbert et al., 2019).

The arrangement of VGCC relative to SVs dictates the speed and efficiency of transmitter release (Wang and Augustine, 2014; Stanley, 2016; Nusser, 2018). In the endbulb, SVs are positioned close to VGCC, resulting in tight coupling that enables rapid and efficient “nanodomain” release upon the arrival of the pre-synaptic AP (Lin et al., 2011). This fast and synchronous SV release is further supported by the expression of the fast calcium sensor protein, synaptotagmin (Syt) 2 (MacLeod and Pandya, 2022). Despite the primarily fast and synchronous SV release, pre-synaptic modulation can still affect the amount and time course of neurotransmitter release. At the endbulb, the activation of pre-synaptic GABA_B_ receptors reduces SV release, thereby influencing information transfer across the synapse (Brenowitz et al., 1998; Brenowitz and Trussell, 2001b; Chanda and Xu-Friedman, 2010).

Specializations for fast and temporally precise signal transmission in the pre-synaptic terminal are complemented by adaptations in the post-synaptic cell to maintain high temporal precision. Several post-synaptic properties are shared between the cells discussed in this review and contribute to efficient encoding of temporal information.

Fast excitatory currents (EPSCs) are primarily mediated by AMPA-type glutamate receptors. Bushy cells express AMPA receptors containing GluA3 and GluA4 subunits (Wang et al., 1998b; Petralia et al., 2000; Ravindranathan et al., 2000; Schmid et al., 2001), with GluA3 subunits clustered at the center of the synapse (Rubio et al., 2017). GluA1 is absent, whereas GluA2 is expressed at low levels (Hunter et al., 1993; Rubio and Wenthold, 1997; Wang et al., 1998b; Gardner et al., 1999, 2001). In mature animals, AMPA receptor subunits are expressed in the *flop splice* variant, which renders them rapidly desensitizing with a fast recovery from desensitization (Sommer et al., 1990; Mosbacher et al., 1994; Geiger et al., 1995; Koike et al., 2000; Gardner et al., 2001; Schmid et al., 2001; Krampfl et al., 2002; Pei et al., 2009). Furthermore, NMDA receptors are present at moderate levels (Watanabe et al., 1994; Gómez-Nieto and Rubio, 2011). The expression of fast-gating AMPA receptors combined with highly synchronous SV release results in very brief EPSCs (<0.5 ms), enabling the synapse to follow high firing rates (Otis and Trussell, 1996; Brenowitz and Trussell, 2001a; Xie and Manis, 2013; Antunes et al., 2020; Zhang et al., 2022).

When stimulated at high frequencies in brain slices, endbulb EPSCs show considerable short-term depression (STD) (Oleskevich et al., 2000; Oleskevich and Walmsley, 2002; Wang and Manis, 2005; Ngodup et al., 2015). This suggests either a high SV release probability, which rapidly depletes the RRP, or post-synaptic receptor desensitization, with the latter having little contribution at the mature endbulb (Brenowitz and Trussell, 2001b; Wang and Manis, 2008). Importantly, under near-physiological low calcium (1–1.5 mM) conditions, short-term depression is reduced and has minimal impact on EPSC amplitudes during ongoing activity (Wang and Manis, 2008; Yang and Xu-Friedman, 2015). These findings align with *in vivo* observations, where the EPSP amplitude remains largely unaffected by preceding events in the presence of spontaneous activity (Kuenzel et al., 2011; Keine et al., 2016; Stasiak et al., 2018). Although EPSC depression levels vary among cells, they are similar across endbulbs that contact the same bushy cell, despite originating from different SGN subtypes (Yang and Xu-Friedman, 2009; Zhang et al., 2022; Wong et al., 2025).

While the generation of APs is controlled by voltage-gated sodium and potassium channels, the cell's intrinsic electrical properties, including input resistance, resting membrane potential, and membrane time constant are influenced by the presence of hyperpolarization-activated cyclic nucleotide–gated (HCN) channels and low-voltage-activated potassium channels (Kv1). In mature animals, bushy cells typically fire one or a few APs at stimulus onset in response to depolarizing square-pulse current injections (Wu and Oertel, 1984; Schwarz and Puil, 1997). This phasic response is due to the expression of dendrotoxin-sensitive, low-threshold, voltage-activated Kv1 channels (Manis and Marx, 1991; Cao et al., 2007), which renders bushy cells suitable for preserving the timing of endbulb inputs (Wu and Oertel, 1984).

Kv1 channels are often expressed alongside HCN channels, which are open at or near the cell's resting membrane potential, thereby reducing the cell's input resistance and shortening the membrane time constant. HCN channels can also enhance temporal precision by facilitating rapid recovery from hyperpolarization (Hassfurth et al., 2009; Cao and Oertel, 2011; Khurana et al., 2011; Golding and Oertel, 2012). Bushy cells express several HCN subtypes (HCN1, HCN2, and HCN4) (Schwarz and Puil, 1997; Leao et al., 2006; Cao et al., 2007; Oertel et al., 2008). HCN1 activates the fastest, rendering it ideally suited to support the demand for high temporal precision (Magee, 1998, 1999; Chen et al., 2001; Moosmang et al., 2001; Khurana et al., 2012; Oertel et al., 2017). Conversely, HCN2 and HCN4 activate more slowly but have a greater potential for modulation via intracellular cAMP (Ludwig et al., 1998, 1999; Santoro et al., 1998; Santoro and Tibbs, 1999).

The combined expression of Kv1 and HCN channels in bushy cells determines their characteristic membrane properties, featuring low input resistance (<100 MΩ) (Wu and Oertel, 1984; Schwarz and Puil, 1997) and fast membrane time constant (<2 ms) (Wang and Manis, 2006; Cao et al., 2007). These properties result in brief EPSPs that minimize temporal summation, allowing only the largest and most synchronous EPSPs to evoke APs (Manis and Marx, 1991; McGinley and Oertel, 2006; Cao et al., 2007).

Finally, rapid repolarization after an AP, which is crucial for enabling high firing rates, is facilitated by high-threshold voltage-activated potassium channels, particularly the Kv3 subfamily (Manis and Marx, 1991; Brew and Forsythe, 1995; Du et al., 1996; Rathouz and Trussell, 1998; Wang and Kaczmarek, 1998; Coetzee et al., 1999; Brown et al., 2016). Bushy cells express Kv3.1, Kv3.3, and Kv3.4, contributing to their brief AP duration (200–500 μs) (Weiser et al., 1995; Li et al., 2001; Pál et al., 2005).

In response to sound, bushy cell firing resembles that of the AN input, displaying a “primary-like” response. Bushy cells exhibit narrow frequency tuning, sharp onsets, and precise encoding of temporal fine structure (Rhode et al., 1983; Rhode and Smith, 1986; Kopp-Scheinpflug et al., 2002). Notably, bushy cells, particularly GBCs, can follow the fine structure of sounds with higher precision than their AN input (Joris et al., 1994a,b; Keine et al., 2016, 2017). Bushy cells are therefore well-suited to preserve and even enhance the temporal precision of the auditory nerve input, enabling them to deliver the fast and temporally precise excitation required by downstream nuclei.

In summary, the first central synapse in the binaural sound localization pathway is formed by a large axosomatic terminal connecting the AN to bushy cells in the cochlear nucleus, which is conserved across many vertebrate species. The large pre-synaptic terminal with multiple AZ and rapid neurotransmitter release, in combination with post-synaptic specializations, such as fast-gating AMPA receptors and intrinsic membrane properties, support rapid and temporally precise action potential generation. These adaptations are fundamental for supporting downstream processing of sound localization.

### The calyx of Held

The MNTB serves as a crucial inhibitory hub within the auditory brainstem. It provides precisely timed glycinergic inhibition to several SOC nuclei, including the LSO, MSO, and superior olivary nucleus (SPN) (Spangler et al., 1985; Banks and Smith, 1992; Kuwabara and Zook, 1992; Wu and Kelly, 1992; Sommer et al., 1993; Srinivasan et al., 2004). This function relies heavily on the calyx of Held—MNTB synapse, which acts as a high-fidelity relay, converting precisely timed excitation from GBCs to an equally precise inhibitory output.

#### Structure and morphology

The GBC-MNTB pathway showcases numerous morphological and physiological specializations designed for fast and reliable information transfer, with the calyx of Held pre-synaptic terminal being the most prominent. This giant excitatory (glutamatergic) terminal is formed by the axons of GBCs located in the contralateral CN and is considered one of the largest pre-synaptic terminals in the mammalian central nervous system (Harrison and Warr, 1962; Morest, 1968). The axons that form the calyx of Held are among the thickest and most heavily myelinated axons in the auditory system, ensuring fast signal conduction (Morest, 1968; Ford et al., 2015). Its exceptional size and experimental accessibility have made the calyx of Held uniquely accessible for electrophysiological experiments, allowing simultaneous patch-clamp recordings from both the pre-synaptic terminal and post-synaptic cell (Forsythe, 1994; Borst et al., 1995), a feat that is currently impossible at smaller conventional synapses. Consequently, the calyx of Held has become the primary model system for studying fundamental processes, such as synaptic transmission, plasticity, and neuronal development (von Gersdorff and Borst, 2002; Schneggenburger and Forsythe, 2006; Borst and Soria van Hoeve, 2012; Yu and Goodrich, 2014; Baydyuk et al., 2016; Neher, 2017; Joris and Trussell, 2018; Sakaba, 2018).

Notably, the size of the MNTB varies between species. It is easily recognizable in rodents and contains a relatively uniform population of large globular cells with tufted dendrites. However, in primates, including humans, the MNTB is poorly developed and appears as a loose collection of cells (Richter et al., 1983; Moore, 1987; Bazwinsky et al., 2003; Kulesza, 2008, 2014; Hilbig et al., 2009; Schmidt et al., 2010; Kulesza et al., 2011; Kulesza and Grothe, 2015). Despite this difference, calyx-type terminals have been identified in both rhesus monkeys and humans, suggesting that this striking morphological specialization offers unique benefits across diverse acoustic environments and is perhaps independent of the prominence or spatial organization of the MNTB (Richter et al., 1983; Bazwinsky et al., 2005; Kulesza, 2014).

#### Organization of synaptic inputs

In contrast to bushy cells, which can receive multiple endbulb inputs, MNTB principal cells are typically contacted by a single glutamatergic calyx terminal, with the occasional occurrence of two or three calyces per MNTB cell (Held, 1893; Vater and Feng, 1990; Rodríguez-Contreras et al., 2006, 2008). This single calyx terminal can engulf more than half of the MNTB cell soma. Similar to endbulbs, calyx axons sprout collateral branches that form conventional boutons on nearby MNTB cells (Lenn and Reese, 1966; Casey and Feldman, 1982; Rodríguez-Contreras et al., 2008; Thomas et al., 2019). In mice, which exhibit a well-developed MNTB, calyx terminals vary in complexity. Larger terminals often exhibit more intricate branching and are highly fenestrated (Grande and Wang, 2011; Grande et al., 2014; Wang et al., 2015; Fekete et al., 2019), presumably to facilitate transmitter diffusion during high-frequency stimulation (Ford et al., 2015). The complex morphology of the calyx includes second- and third-order branches consisting of swellings and finger-like stalks. These structures may comprise distinct biochemical compartments (Rowland et al., 2000; Taschenberger et al., 2002; Wimmer et al., 2006; Spirou et al., 2008) with different synaptic vesicle release probabilities; however, their physiological functions are currently poorly understood (Grande and Wang, 2011; Fekete et al., 2019).

Similar to the endbulb, the calyx harbors hundreds (500–1,000) of AZs (Sätzler et al., 2002; Taschenberger et al., 2002; Wimmer et al., 2006; Dondzillo et al., 2010). Although large overall, each individual AZ is morphologically similar to conventional bouton synapses (Rowland et al., 2000; Sätzler et al., 2002; Taschenberger et al., 2002; Lin et al., 2011; Schneggenburger et al., 2012; Thomas et al., 2019). The sheer size of the pre-synaptic terminal and the number of release sites result in an enormous RRP, exceeding 1,000 SVs (Schneggenburger et al., 1999; Wu and Borst, 1999; Bollmann et al., 2000; Sun and Wu, 2001; Sätzler et al., 2002; de Lange et al., 2003; Wimmer et al., 2006; Lin et al., 2011). Mirroring the endbulb, a dense network of mitochondria has been described for the calyx, presumably serving a similar function in supporting the metabolic demand and acting as an additional calcium buffer (Lenn and Reese, 1966; Nakajima, 1971; Rowland et al., 2000; Wimmer et al., 2006; Thomas et al., 2019).

#### Pre- and post-synaptic physiology

As perhaps the largest pre-synaptic terminal in the mammalian central nervous system (CNS), the calyx of Held has been instrumental in deciphering much of what we know about neurotransmitter release. Similar to the endbulb of Held, pre-synaptic APs are brief (~200 μs halfwidth) (Taschenberger et al., 2002). This brevity is supported by the exclusion of sodium channels from the calyx terminal. Instead, they are densely clustered at the last elongated axonal heminode (Leão et al., 2005). The calyx expresses various potassium channels: Low-threshold channels are mainly composed of Kv1.2 homomers, with some Kv1.3. Kv1.3 is located at the calyx terminal (Gazula et al., 2010), whereas Kv1.1 and Kv1.2 are excluded from the terminal and concentrated at the axon-terminal transition zone, likely to reduce nerve terminal excitability (Dodson et al., 2003; Ishikawa et al., 2003). High-threshold Kv3 potassium channels help rapidly repolarize the membrane, shortening AP duration, thereby regulating transmitter release and enabling high firing rates (Taschenberger and von Gersdorff, 2000; Joshi and Wang, 2002; Chao and Yang, 2019; Richardson et al., 2022). This specific arrangement of sodium and potassium channels ensures that the pre-synaptic AP remains remarkably stable, even during high firing rates exceeding several hundred Hertz (Wang and Kaczmarek, 1998; Taschenberger and von Gersdorff, 2000; Sierksma and Borst, 2017).

Similar to the endbulb, calcium entry into the mature calyx is exclusively mediated by Ca_V_2.1 channels (Doughty et al., 1998; Iwasaki and Takahashi, 1998; Lin et al., 2011). Calcium currents in the calyx seem to inactivate more strongly during step-like depolarization than those in the endbulb (Forsythe et al., 1998; Lin et al., 2011). However, during AP-like depolarizations, VGCC inactivation may be negligible, and calcium current facilitation may play a dominant role, especially in the endbulb (Wang and Kaczmarek, 1998; Felmy et al., 2003; Inchauspe et al., 2007; Müller et al., 2008, 2010; Lin et al., 2011).

In the mature calyx, SVs are positioned closely (~20 nm) to VGCCs, enabling rapid and efficient nanodomain release upon the arrival of the pre-synaptic AP (Taschenberger and von Gersdorff, 2000; Taschenberger et al., 2002; Fedchyshyn and Wang, 2005; Renden and von Gersdorff, 2007; Wang et al., 2008; Kochubey et al., 2009; Eggermann et al., 2011; Chen et al., 2015; Stanley, 2016). The expression of the fast calcium sensor Syt2 further supports rapid SV release (Pang et al., 2006; Cooper and Gillespie, 2011; Kochubey et al., 2016). Recently, Syt3 was identified as the calcium sensor responsible for efficient SV replenishment at the calyx of Held (Weingarten et al., 2022), enabling fast and indefatigable synaptic transmission.

Early slice studies suggested a high SV release probability at the calyx, given the large post-synaptic currents (Borst and Sakmann, 1996). However, studies performed under more physiological conditions (body temperature, low calcium) indicate that the release probability at the calyx is low (Lorteije et al., 2009; Borst, 2010; Keine et al., 2022). Adding to this complexity, some findings suggest that SV release probabilities can differ between calyces and even between AZ within the same calyx, indicating functional heterogeneity (Grande and Wang, 2011; Fekete et al., 2019). Explaining the diverse forms of short-term plasticity observed under different conditions requires models containing multiple SV pools with sequential priming processes and distinct release probabilities (Sakaba, 2006, 2018; Wölfel et al., 2007; Guo et al., 2015; Neher, 2015; Lin et al., 2022, 2025). However, during physiological spontaneous activity, relatively simple models are often sufficient to approximate the calyx' short-term plasticity (Hermann et al., 2007, 2009).

Although SV release is primarily fast and synchronous, it can be modulated by the activation of pre-synaptic receptors, including GABA_B_ and glycine receptors (Turecek and Trussell, 2001; Price and Trussell, 2006), as well as intracellular signaling molecules such as cAMP (Kaneko and Takahashi, 2004).

On the post-synaptic side, AMPA receptors are evenly distributed throughout the post-synaptic density (Budisantoso et al., 2013), similar to other glutamatergic synapses (Budisantoso et al., 2012). These receptors primarily consist of GluA4 in the fast *flop* variant (Geiger et al., 1995; Hermida et al., 2010; Yang et al., 2011). Most AMPA receptors also include at least one GluA2 subunit, rendering them impermeable to calcium (Koike-Tani et al., 2005; Lujan et al., 2019). Similar to bushy cells, NMDA receptors are expressed at moderate levels in adult animals but contribute little to the large and fast synaptic currents at this synapse (Barnes-Davies and Forsythe, 1995; Wang and Kaczmarek, 1998; Sato et al., 1999; Nakagawa et al., 2000; Futai et al., 2001).

The resulting EPSCs in MNTB neurons are fast (<0.5 ms) and display mature characteristics immediately after hearing onset (~P12 in mice) (Taschenberger and von Gersdorff, 2000; Futai et al., 2001; Joshi and Wang, 2002; Fedchyshyn and Wang, 2005; Keine et al., 2022). Under near-physiological conditions, high-frequency electrical stimulation of the calyx axon causes initial EPSC facilitation, followed by depression, consistent with a low release probability *in vivo* (von Gersdorff and Borst, 2002; Lorteije et al., 2009; Borst, 2010; Keine et al., 2022; Kladisios et al., 2023). Similar to the endbulb-bushy cell synapse, the impact of synaptic short-term plasticity appears to be subtle *in vivo*, with only modest EPSP facilitation at brief intervals (Lorteije et al., 2009; Crins et al., 2011; Sonntag et al., 2011; Stasiak et al., 2018). Receptor desensitization contributes little to short-term depression because of the low SV release probability and rapid glutamate clearance from the highly fenestrated calyx (Joshi and Wang, 2002; Taschenberger et al., 2002, 2005; Koike-Tani et al., 2008). Notably, short-term depression in mice is inversely correlated with calyx complexity: simple calyces with higher release probability show strong EPSC depression and reduced AP firing reliability compared to more complex calyces (Grande and Wang, 2011; Fekete et al., 2019).

The combination of an elaborate pre-synaptic terminal, large SV pools, and efficient release mechanisms ensures strong excitation of the MNTB cell, leading to reliable AP generation even during prolonged activity. Although the delay between the pre-synaptic and post-synaptic AP can increase considerably during sustained high-frequency stimulation, most of this added delay occurs between the EPSP and AP (Guinan and Li, 1990; Elezgarai et al., 2003; Fedchyshyn and Wang, 2007; Mc Laughlin et al., 2008; Tolnai et al., 2009). The delay between the pre-synaptic AP and EPSP remains remarkably stable in mature animals, emphasizing the efficiency of SV release and replenishment in the calyx (Mc Laughlin et al., 2008; Sonntag et al., 2011). Notably, neurons tuned to low sound frequencies tend to exhibit more stable transmission delays, suggesting a specialization for activity-invariant timing of synaptic transmission (Stange-Marten et al., 2017).

Post-synaptic intrinsic properties further shape the MNTB output. Unlike bushy cells, which predominantly express HCN1, MNTB neurons mainly express HCN2 but not HCN1 (Koch et al., 2004; Leao et al., 2006; Khurana et al., 2012; Baumann et al., 2013; Kopp-Scheinpflug et al., 2015). This difference in HCN subtype expression might be related to the cell's function: bushy cells might benefit from the fast HCN1 dynamics to enhance temporal precision for coincidence detection, whereas MNTB neurons, receiving input from a single suprathreshold calyx terminal, do not require coincident inputs to generate APs.

The combined expression of Kv1 channels (Brew and Forsythe, 1995; Dodson et al., 2002; Kladisios et al., 2023) and HCN channels results in an input resistance (~100 MΩ) (Banks and Smith, 1992; Hassfurth et al., 2009; Kladisios et al., 2020, 2023) and membrane time constant (2–4 ms) (Scott et al., 2005; Roberts et al., 2014; Kladisios et al., 2020, 2023) comparable to bushy cells. Consequently, depolarizing square-pulse current injections result in a single AP in adult MNTB cells. This phasic firing is important for limiting the number of APs and preserving temporal precision in response to the large EPSCs generated by the calyx terminal. Fast repetitive firing of MNTB neurons is supported by the expression of Kv3.1 and Kv3.3 channels (Wang et al., 1998a; Choudhury et al., 2020).

The single suprathreshold calyx in combination with an MNTB cell that responds with a single, well-timed AP leads to MNTB activity that is a faithful representation of its GBC input. Consequently, MNTB responses to sound are mostly indistinguishable from their GBC input (Mc Laughlin et al., 2008; Englitz et al., 2009; Sonntag et al., 2011) (but see *Functional consequences in relation to synaptic specializations*).

In summary, the calyx of Held—MNTB synapse combines several morphological and physiological specializations, rendering it an ideal relay station to provide well-timed and sustained inhibition to several nuclei in the auditory brainstem, including the two binaural nuclei involved in sound localization, the MSO and LSO.

### Medial superior olive

The MSO is the first station in the auditory system where differences between the arrival times of sounds at both ears (interaural time differences, ITD) are computed. Cellular and synaptic specializations enable MSO neurons to detect these time differences with sub-millisecond precision (Goldberg and Brown, 1969; Yin and Chan, 1990). While it is generally accepted that MSO neurons act as coincidence detectors, the exact mechanisms underlying their extraordinary precision, including the origin of internal delays and the role of inhibition, are still actively debated.

#### Structure and morphology

The MSO has been described in many mammals, but its size and cellular organization vary considerably across species. Generally, it tends to be larger and more organized in animals with large heads and sensitive low-frequency hearing, which rely on ITDs for sound localization (Harrison and Irving, 1966a; Irving and Harrison, 1967; Moore and Moore, 1971; Glendenning and Masterton, 1998; Grothe, 2000; Grothe and Pecka, 2014). Various studies have identified up to four distinct cell types in the MSO of different animals, but the most common are bipolar cells, often referred to as principal cells (Schwartz, 1977; Kiss and Majorossy, 1983; Henkel and Brunso-Bechtold, 1990; Smith, 1995; Kulesza, 2007). These cells have a characteristic spindle-like shape, with thick, tapered dendrites (~4 μm diameter, 100–200 μm length) extending from the medial and lateral poles of the cell soma (Stotler, 1953; Kiss and Majorossy, 1983; Henkel and Brunso-Bechtold, 1990; Kudo et al., 1990; Smith, 1995; Rautenberg et al., 2009). In species with sensitive low-frequency hearing that effectively use ITDs for sound localization (e.g., gerbils, cats, and humans), these bipolar cells are arranged in a single column and aligned in the parasagittal plane, with their dendrites extending medially and laterally (Schwartz, 1977; Kulesza, 2007; Rautenberg et al., 2009; Bondy et al., 2021). Conversely, in animals that do not rely heavily on ITDs (e.g., bats, mice, rats, and opossums), the MSO is still present, but the organization of cell bodies, dendrites, and synaptic inputs is less strictly defined, suggesting that it might serve functions other than primary ITD processing in these species (Ollo and Schwartz, 1979; Grothe and Park, 2000; Kapfer et al., 2002; Fischl et al., 2016; Rincón et al., 2024).

MSO neurons are excitatory and project primarily to the auditory midbrain, the ipsilateral IC, and the dorsal and intermediate nuclei of the lateral lemnicus (DNLL and INLL, respectively) (Glendenning et al., 1981; Henkel and Spangler, 1983; Casseday et al., 1988; Vater et al., 1995; Henkel, 1997; Oliver et al., 2003; Loftus et al., 2004; Cant, 2013; Rincón et al., 2024).

#### Organization of synaptic inputs

In contrast to the giant endbulb and calyx terminals discussed earlier, synaptic inputs onto MSO neurons are smaller conventional bouton-type terminals. Nevertheless, the soma and proximal dendrites of MSO neurons are densely covered with these inputs (Perkins, 1973; Lindsey, 1975; Schwartz, 1977).

MSO cells receive both excitatory (glutamatergic) inputs originating from SBCs in the ipsi- and contralateral CN (Stotler, 1953; Warr, 1966; Osen, 1969b; Lindsey, 1975; Grothe and Sanes, 1993; Kitzes et al., 1995; Smith, 1995; Magnusson et al., 2005; Scott et al., 2005; Chirila et al., 2007) and inhibitory (glycinergic) inputs primarily from the MNTB, with a smaller contribution from the LNTB, relaying information reflecting the activity from the contralateral ear (Adams and Mugnaini, 1990; Cant and Hyson, 1992; Kuwabara and Zook, 1992; Spirou and Berrebi, 1997; Spirou et al., 1998; Roberts et al., 2014). Morphological and electrophysiological evidence suggests that the number of individual fiber inputs is surprisingly low for a coincidence detector, ranging between 2–4 fibers for inhibition and 4–8 fibers for excitation (Couchman et al., 2010).

A key feature in animals that use ITDs is the precise spatial segregation of these inputs (Stotler, 1953; Clark, 1969a; Kuwabara and Zook, 1992; Kapfer et al., 2002). Excitatory inputs are distributed along the dendrites (Clark, 1969b; Callan et al., 2021), with ipsilateral and contralateral inputs confined to the lateral and medial dendrites, respectively (Stotler, 1953; Clark, 1969b; Lindsey, 1975; Smith et al., 1993). Despite their small size, a single excitatory axon forms multiple points of contact along its target dendrite, each containing multiple active zones (Clark, 1969b; Lindsey, 1975; Kiss and Majorossy, 1983; Brunso-Bechtold et al., 1990; Kapfer et al., 2002; Callan et al., 2021). In contrast, inhibitory inputs are small and confined to the soma and proximal dendrites (Stotler, 1953; Clark, 1969b; Perkins, 1973; Kuwabara and Zook, 1992; Grothe and Sanes, 1993, 1994; Kapfer et al., 2002; Werthat et al., 2008; Grothe et al., 2010; Couchman et al., 2012). Importantly, this strict segregation of dendritic excitation and somatic inhibition is less apparent or absent in species with poor low-frequency hearing that do not use ITDs. This suggests that the spatial arrangement of synaptic inputs is a crucial adaptation for enhancing ITD detection in the sub-millisecond range required for sound localization (Kapfer et al., 2002; Seidl and Grothe, 2005).

#### Pre- and post-synaptic physiology

Owing to their small size and relative inaccessibility for direct patch-clamp recordings, the calcium dynamics and SV release mechanisms in these bouton inputs remain largely unexplored. However, some key features have been uncovered: glycine release is mediated by Ca_V_2.1 channels with some contribution from Ca_V_2.2 (N-type), whereas glutamate release is mediated by both Ca_V_2.1 and Ca_V_2.2, at least in young rats around hearing onset (Barnes-Davies et al., 2001). Consistent with the MSO's need for precise timing, pre-synaptic terminals on MSO neurons express Syt2, enabling fast and synchronous SV release, similar to the endbulb and calyx (Cooper and Gillespie, 2011). Both excitatory and inhibitory inputs can be modulated by the activation of pre-synaptic GABA_B_ receptors, at least in the juvenile MSO (Hassfurth et al., 2010; Stange et al., 2013). Post-synaptically, MSO principal cells are equipped with AMPA receptors that are distributed across the soma and dendrites, apparently with little subunit preference (Caicedo and Eybalin, 1999), whereas glycine receptors are concentrated primarily on the soma (Couchman et al., 2012). NMDA receptors are expressed at moderate levels during adulthood; however, their functional role remains unclear (Smith et al., 2000; Couchman et al., 2012). Despite originating from smaller pre-synaptic terminals, both excitatory and inhibitory synaptic currents are surprisingly large, comparable in amplitude to those evoked by the much larger endbulb (Couchman et al., 2010; Franzen et al., 2015), and show comparable short-term depression during high-frequency stimulation (Couchman et al., 2010; Fischl et al., 2016). Crucially, their kinetics are dramatically different: EPSCs are extremely fast, with decay times of approximately 250 μs, whereas IPSCs are several-fold slower (~1.5 ms decay) (Magnusson et al., 2005; Couchman et al., 2010; Myoga et al., 2014; Franzen et al., 2020).

While the pre-synaptic physiology and synaptic currents share some features with the larger endbulb and calyx terminals, the intrinsic membrane properties of MSO neurons are exceptionally tuned for microsecond coincidence detection and set them apart from most other auditory brainstem neurons. MSO neurons exhibit a remarkably low input resistances (<10 MΩ) and extraordinarily fast membrane time constants (<300 μs)—about 10 times faster than those of MNTB neurons (Magnusson et al., 2005; Scott et al., 2005; Chirila et al., 2007; Couchman et al., 2010; Fischl et al., 2016; Nabel et al., 2019; Franzen et al., 2020; Kladisios et al., 2020; Siveke et al., 2021). These rapid membrane dynamics ensure that EPSPs are very brief (<1 ms) (Kullmann et al., 2005; Mathews et al., 2010; Winters et al., 2017), minimizing the time window for input summation and enhancing sensitivity to coincident events. These extraordinary membrane properties arise largely from the expression of Kv1 and HCN1 channels (Svirskis et al., 2002; Koch et al., 2004; Scott et al., 2005; Mathews et al., 2010; Khurana et al., 2012; Baumann et al., 2013; Kopp-Scheinpflug et al., 2015; Fischl et al., 2016; Winters et al., 2017). In addition to contributing to the fast membrane time constant, the expression of Kv1 channels may also facilitate resonance behavior up to several hundred Hertz, potentially further enhancing ITD detection (Remme et al., 2014; Mikiel-Hunter et al., 2016; Fischer et al., 2018). Notably, Kv1 channels are gradually expressed along the dendrites, ensuring that EPSPs arriving at different dendritic locations produce similar voltage changes at the soma. Consequently, inputs from all over the dendritic tree can contribute effectively to ITD computation (Mathews et al., 2010; Winters et al., 2017).

In MSO neurons, the integration of synaptic inputs is finely tuned to detect ITDs. Despite individual EPSCs being relatively large, the cell's low input resistance limits EPSP amplitudes, often rendering them insufficient to evoke APs. Consequently, triggering APs requires the near-simultaneous arrival of multiple excitatory inputs (van der Heijden et al., 2013; Franken et al., 2015; Plauška et al., 2016; Kladisios et al., 2020).

Although these EPSPs sum largely linearly (Roberts et al., 2013; van der Heijden et al., 2013; Franken et al., 2015; Plauška et al., 2016), the generation of an AP depends non-linearly on the resulting EPSP amplitude (van der Heijden et al., 2013). A notable consequence of their extremely low input resistance is that MSO neurons typically generate small somatic APs (often <10 mV), rendering *in vivo* recordings with high signal-to-noise ratios particularly challenging (Yin and Chan, 1990; Scott et al., 2005; Chirila et al., 2007; Couchman et al., 2010; van der Heijden et al., 2013; Franken et al., 2015).

MSO responses *in vivo* are based on the coincidence detection of binaural inputs; thus, firing rates vary with ITD (Goldberg and Brown, 1969; Yin and Chan, 1990; Pecka et al., 2008; van der Heijden et al., 2013; Franken et al., 2015; Plauška et al., 2016). Principal cells in the MSO favor low-frequency sounds, and their fast membrane time constant and rapid EPSCs allow them to detect microsecond differences in the arrival time between inputs from both ears. Notably, MSO neurons are generally broadly tuned, and their firing rates are modulated over ITD ranges that exceed the “physiological ITD” based on the physical difference between the two ears (Brand et al., 2002; Pecka et al., 2008).

In summary, MSO neurons are highly specialized coincidence detectors, fine-tuned for temporal precision, supported by their bipolar morphology, rapid synaptic signaling, and exceptionally fast membrane properties. These adaptations allow them to calculate ITDs with the microsecond accuracy required for sound localization.

### Lateral superior olive

The LSO is a key component of the ILD pathway. The auditory system can distinguish ILDs as small as 1 dB, which corresponds to a difference in sound location of 1° in the horizontal plane (Mills, 1958). This remarkable acuity arises primarily from LSO neurons that compare ipsilateral excitation with contralateral inhibition. LSO neurons then provide ascending projections to regions in the IC similar to those targeted by the MSO, but with excitatory neurons targeting the IC bilaterally and inhibitory neurons projecting ipsilaterally (Oliver et al., 1995; Loftus et al., 2004; Haragopal and Winters, 2023).

#### Structure and morphology

Similar to the MSO, the LSO is a common structure observed in all terrestrial mammals studied to date (Glendenning and Masterton, 1998; Tollin, 2003; Grothe et al., 2010; Grothe and Pecka, 2014). However, its overall shape and number of neurons can differ significantly across species (Irving and Harrison, 1967; Willard and Martin, 1983; Sanes et al., 1989; Casey, 1990; Moore et al., 1995; Moore, 2000; Kulesza, 2007; Hilbig et al., 2009; Reuss et al., 2009; Sterenborg et al., 2010; Hirtz et al., 2011; 2012; Rosengauer et al., 2012; Bazwinsky-Wutschke et al., 2016; Nothwang, 2016). Up to seven different cell types have been identified in the LSO, with principal cells being the most abundant, comprising approximately 75% of the neuronal population (Ollo and Schwartz, 1979; Helfert and Schwartz, 1986, 1987; Rietzel and Friauf, 1998; Kulesza, 2007; Franken et al., 2018). For the remainder of this review, we will focus on LSO principal cells, which possess a spindle-shaped soma with two prominent primary dendrites emerging from opposite somatic poles (Cant, 1984; Helfert and Schwartz, 1986, 1987; Schofield and Cant, 1991; Rietzel and Friauf, 1998), resembling MSO principal neurons. In contrast to the thick MSO dendrites, LSO dendrites are more intricate and branch extensively, spreading across a considerable rostrocaudal distance (Scheibel and Scheibel, 1974; Helfert and Schwartz, 1986).

#### Organization of synaptic inputs

LSO principal neurons receive their main excitatory inputs from SBCs in the ipsilateral CN and their primary inhibitory inputs from the contralateral CN via GBCs relayed through the MNTB (Moore and Caspary, 1983; Smith et al., 1993; Sommer et al., 1993). Additionally, excitatory inputs from GBCs and planar multipolar neurons in the ipsilateral VCN have been reported (Friauf and Ostwald, 1988; Smith et al., 1991; Doucet and Ryugo, 2003).

Similar to the MSO, a substantial portion of the LSO principal cell soma is covered with synaptic inputs (Helfert and Schwartz, 1986; Franken et al., 2018), which are predominantly inhibitory (Cant, 1984; Wenthold et al., 1987; Helfert et al., 1989; Friauf, 1992; Helfert et al., 1992; Brunso-Bechtold et al., 1994; Cooper and Gillespie, 2011; Hirtz et al., 2012; Gjoni et al., 2018a). Inhibitory and excitatory inputs are somewhat spatially segregated; inhibitory terminals tend to cluster on or near the soma, whereas excitatory terminals are located more distally on thin dendrites (Cant, 1984; Helfert et al., 1992; Smith et al., 1998). Inhibitory dominance is also reflected in synaptic morphology. Although both inputs form conventional boutons, the inhibitory terminals are notably larger and more complex. Specifically, individual inhibitory axons form multiple large swellings or varicosities (~6, range 1–13), each typically containing several AZs (~3, range 1–11). This structural arrangement provides the basis for strong unitary inhibition from an estimated 4–8 converging fibers, totaling hundreds of AZs (Noh et al., 2010; Gjoni et al., 2018a,b). In contrast, the more numerous excitatory fibers (~20–40 fibers) primarily target dendrites and form smaller terminals with few varicosities (1–3) that usually contain only a single AZ (Gjoni et al., 2018a,b).

#### Pre- and post-synaptic physiology

While direct electrophysiological recordings of LSO pre-synaptic terminals are currently lacking, several studies have revealed similarities with other auditory brainstem synapses. Synaptic transmission at both excitatory (glutamatergic) and inhibitory (glycinergic) terminals appears to rely predominantly on Ca_V_2.1 calcium channels and Syt1/2, ensuring rapid and synchronous SV release (Alamilla and Gillespie, 2011; Giugovaz-Tropper et al., 2011; Bouhours et al., 2017), similar to the endbulb and calyx of Held.

Pre-synaptic modulation mirrors the patterns observed elsewhere in the circuit. Activation of pre-synaptic GABA_B_ receptors reduces SV release from both excitatory and inhibitory inputs. Notably, this modulation appears to have a stronger effect on excitatory inputs, suggesting a potential mechanism for dynamically adjusting ILD sensitivity by preferentially dampening excitation (Magnusson et al., 2008).

On the post-synaptic side, glutamatergic transmission is mediated by AMPA receptors containing primarily GluA2/3 and GluA4 subunits, following the general pattern observed in the auditory brainstem (Caicedo and Eybalin, 1999; Schwartz and Eager, 1999; Schmid et al., 2001). Similar to other SOC nuclei, mature LSO neurons express moderate levels of NMDA receptors, but their functional role remains unclear (Munemoto et al., 1998; Sato et al., 1999; Nakagawa et al., 2000).

Inhibition is mediated by abundant glycine receptors clustered on the soma and proximal dendrites (Friauf et al., 1997; Sato et al., 2000; Kapfer et al., 2002). A unique feature of inhibition in the LSO is its remarkable speed: inhibitory currents decay rapidly (~0.7 ms), essentially matching the speed of excitatory currents (Moore and Caspary, 1983; Wu and Kelly, 1991, 1992; Kandler and Friauf, 1995; Wu and Kelly, 1995; Kotak et al., 1998; Pilati et al., 2016). This contrasts with most other brainstem nuclei, where inhibition is typically several times slower than excitation (Smith et al., 2000; Couchman et al., 2010; Xie and Manis, 2013; Mayer et al., 2014; Nerlich et al., 2014a,b; Rajaram et al., 2019).

The combination of large inhibitory terminals with multiple release sites and abundant post-synaptic glycine receptors generates strong inhibition (Gjoni et al., 2018a) with only moderate short-term depression over several seconds (Giugovaz-Tropper et al., 2011; Walcher et al., 2011; Kramer et al., 2014; Krächan et al., 2017). This sustained SV release is supported by rapid and efficient SV recovery mechanisms, rendering these synapses fatigue-resistant and capable of maintaining high temporal precision during ongoing activity (Kramer et al., 2014; Krächan et al., 2017; Brill et al., 2019). Excitatory inputs, while producing smaller EPSCs, match this reliability with comparable SV replenishment rates (Case et al., 2011; Garcia-Pino et al., 2017; Gjoni et al., 2018b; Brill et al., 2019).

Post-synaptic specializations further enhance temporal precision in the LSO. Principal neurons exhibit low input resistance (~50 MΩ) and a fast membrane time constant (1–3 ms) (Hassfurth et al., 2009; Sterenborg et al., 2010; Walcher et al., 2011; Haragopal and Winters, 2023; Maraslioglu-Sperber et al., 2024), supported by the expression of Kv1 (Barnes-Davies et al., 2004) and HCN1/2 channels (Koch et al., 2004; Leao et al., 2006; Kopp-Scheinpflug et al., 2015). While not as “leaky” as MSO neurons, LSO principal neurons have significantly faster membrane dynamics than bushy cells and MNTB neurons. The specific placement of HCN channels along the dendrites can further modulate EPSP integration by locally decreasing input resistance (Leão et al., 2011).

While other neurons discussed in this review typically respond to depolarizing square-pulse current injections with one or a few APs at the onset (Wu and Oertel, 1984; Wu and Kelly, 1991; Brew and Forsythe, 1995; Smith, 1995; Scott et al., 2005; Caspari et al., 2015; Franzen et al., 2015; Kladisios et al., 2020), LSO can either fire single APs or exhibit sustained AP firing (Tsuchitani, 1988; Finlayson and Caspary, 1991; Wu and Kelly, 1991; Barnes-Davies et al., 2004; Sterenborg et al., 2010; Winters et al., 2017; Maraslioglu-Sperber et al., 2024). The physiological roles of these different types of principal neurons and their contributions to ILD coding are not yet fully understood.

As coincidence detectors integrating ipsilateral excitation and contralateral inhibition (relayed via the MNTB), LSO neurons crucially depend on the precise relative timing between these opposing inputs. *In vivo*, this timing is remarkably matched: excitatory and inhibitory signals arrive at the LSO nearly simultaneously, despite the inhibitory pathway being longer and including an additional synapse (Finlayson and Caspary, 1989; Joris and Yin, 1998; Tollin and Yin, 2005). This latency matching is achieved through the specializations of the GBC-MNTB pathway discussed previously, namely the large diameter of GBC axons and the exceptionally fast synaptic transmission at the calyx of Held—MNTB junction. LSO principal neurons favor high-frequency sounds, and the integration of ipsilateral excitation and contralateral inhibition results in firing rates that vary with ILD. Responses to ipsilateral (“excitatory”) sounds have long been characterized as “chopper”-type (Guinan et al., 1972; Tsuchitani, 1977; Finlayson and Caspary, 1989; Joris and Yin, 1995; Tollin et al., 2008; Tsai et al., 2010; Greene and Davis, 2012). However, this characterization has recently been challenged by *in vivo* patch-clamp recordings, suggesting that LSO principal cells, at least in the ventral LSO of the Mongolian gerbil, are similar to MSO cells, exhibiting onset responses (Franken et al., 2018). Further highlighting the significance of timing, the same study found that changing ILDs affects the latency of the inhibitory input more profoundly than its amplitude (Franken et al., 2018). Intriguingly, LSO neuron firing seems to be facilitated when inhibition slightly precedes excitation (Irvine et al., 2001; Beiderbeck et al., 2018), underscoring the notion that precise temporal integration is vital even within this nominal intensity-processing pathway.

## The octopus cell—VNLL pathway

While the bushy cell—superior olive pathway is primarily associated with binaural computation and detection of interaural time and level differences, the parallel octopus cell—VNLL pathway is thought to be crucial for monaural spectrotemporal cues. These include the detection of onsets and gaps, which are important features for vocalization, speech understanding, and auditory scene analysis.

Octopus cells located in the caudal and ventral portion of the VCN receive auditory nerve input and project to several targets, including the VNLL. The VNLL, an elongated nucleus between the SOC and the dorsal NLL, receives major inputs from the CN, MNTB, and periolivary nuclei (Glendenning et al., 1981; Spangler et al., 1985). Functionally, VNLL neurons are predominantly inhibitory (glycine and/or GABA) and provide a major source of inhibition to the contralateral inferior colliculus (IC) and other targets in the NLL and SOC (Brunso-Bechtold et al., 1981; Zook and Casseday, 1982, 1987; Willard and Martin, 1983; Whitley and Henkel, 1984; Tanaka et al., 1985; Vater et al., 1992; Sommer et al., 1993; Saint Marie et al., 1997; Schofield and Cant, 1997; Ueyama et al., 1999; Riquelme et al., 2001; Kelly et al., 2009; Moore and Trussell, 2017). Similar to bushy cells and MNTB cells, globular cells in the VNLL's ventral pole receive large axosomatic pre-synaptic terminals (“VNLL endbulbs”) originating from octopus cells of the contralateral CN (Friauf and Ostwald, 1988; Vater and Feng, 1990; Schofield, 1995; Adams, 1997; Schofield and Cant, 1997; Vater et al., 1997; Smith et al., 2005).

### Octopus cells

Octopus cells within the CN are specialized neurons that detect the precise timing of complex acoustic events, particularly those with broad frequency content occurring simultaneously. By integrating information across many auditory nerve fibers from a wide range of frequencies on a sub-millisecond timescale, they can signal the precise timing of these events to higher auditory centers.

#### Structure and morphology

Located in the most posterior and dorsal part of the VCN, most octopus cells possess a defining anatomical feature: several thick dendrites emerge primarily from one side of the cell body and extend across the frequency axis of auditory nerve fibers, resembling the tentacles of an octopus (Harrison and Irving, 1966b; Osen, 1969b). This distinct morphology is conserved across species with diverse hearing ranges, including cats (Osen, 1969b; Kane, 1973), gerbils (Cant and Benson, 2006; Bazwinsky et al., 2008), rats (Alibardi, 2003), chinchillas (Feng et al., 1994; Ostapoff et al., 1994), mice (Felix et al., 2017), bats (Vater and Feng, 1990), and humans (Adams, 1986, 1997; Kulesza, 2014). A crucial aspect of their morphology is the orientation of their dendrites, which stretch perpendicular to the tonotopically organized AN endings. This arrangement allows a single octopus cell to receive inputs from numerous auditory nerve fibers carrying a wide band of frequencies, covering approximately 1/3 of the animal's hearing range (Harrison and Warr, 1962; Harrison and Irving, 1966b; Golding et al., 1995; Oertel et al., 2000). Similar to GBCs, octopus cells are excitatory (glutamatergic) and possess exceptionally large-diameter axons, ensuring the rapid conduction of their output signals (Rhode et al., 1983; Oertel et al., 1990; Schofield, 1995; Schofield and Cant, 1997). In some species, such as mice and cats, octopus cells cluster together to form a sharply defined “octopus cell area” that is almost exclusively populated by these neurons (Osen, 1969b; Wickesberg et al., 1994; Golding et al., 1995, 1999).

#### Organization of synaptic inputs

Octopus cells receive their primary excitatory input directly from the AN (Harrison and Irving, 1966b; Kane, 1973). Unlike the giant endbulb terminals on bushy cells, these inputs are smaller, more numerous bouton-type synapses (Alibardi, 2003). A single octopus cell integrates inputs from many (>60) individual AN fibers (De No, 1933; Brown and Ledwith, 1990; Cao and Oertel, 2010), primarily originating from low-threshold type 1a SGNs with short latency and low temporal jitter (Kreeger et al., 2025). These inputs appear to be spatially organized along the dendrites: ANFs sensitive to low sound frequencies tend to synapse closer to the soma and proximal dendrites, whereas fibers sensitive to higher sound frequencies target more distal dendritic regions (Harrison and Irving, 1966b; Golding et al., 1995; Oertel et al., 2000).

Although some larger boutons make contact with the cell soma (Kane, 1973; Alibardi, 2003), the vast majority (>80%) of excitatory synapses are located on the dendrites, highlighting the importance of dendritic integration for octopus cell computation (Kreeger et al., 2025). Anatomical evidence and data obtained from *in vivo* whole-cell recordings suggest that excitatory inputs are of different sizes, creating a bias toward certain frequencies (Kane, 1973; Alibardi, 2003; Lu et al., 2022).

In addition to this massive excitatory drive, octopus cells receive inhibitory inputs that primarily target dendrites, which may shape the timing and magnitude of incoming excitatory signals and thus influence neuronal computation (Adams and Mugnaini, 1987; Saint Marie et al., 1989; Juiz et al., 1996; Alibardi, 2003; Kreeger et al., 2025). However, the origin, tonotopic arrangement, and physiological role of these inhibitory inputs remain largely unknown (Kreeger et al., 2025).

#### Pre- and post-synaptic physiology

The unique structure and input organization dictate the specialized function of octopus cells, enabling them to act as monaural coincidence and sweep detectors with sub-millisecond precision (Oertel, 1983; Golding et al., 1995; Golding and Oertel, 2012; Lu et al., 2022). Their extraordinary temporal acuity arises from several key biophysical adaptations. Similar to MSO neurons, octopus cells possess a very “leaky” membrane, resulting in an extremely low input resistance (<10 MΩ) and a remarkably fast membrane time constant (~200 μs). This leakiness is primarily due to the dense expression of Kv1 and HCN channels (Golding et al., 1999; Bal and Oertel, 2000, 2001; Cao and Oertel, 2005; Oertel et al., 2008). The HCN-mediated current is particularly large in octopus cells and activated at more depolarized membrane potentials than in other brainstem neurons, leaving a substantial number of these channels open at rest (Golding et al., 1995, 1999; Cao and Oertel, 2011).

Post-synaptically, octopus cells express fast-gating AMPA receptors (Golding et al., 1995; Hackney et al., 1996) that lack the GluA2 subunit, making them permeable to calcium, and resulting in large synaptic currents (Gardner et al., 1999, 2001). The combination of the fast membrane time constant and these fast-gating AMPA receptors leads to very brief EPSPs, effectively preventing temporal summation unless events arrive in near-perfect synchrony (Oertel, 1983; Golding et al., 1995, 1999; McGinley and Oertel, 2006; Cao and Oertel, 2011, 2017). Similar to other auditory brainstem neurons, octopus cells express Kv3.1 potassium channels, which minimize AP duration (~300 μs) and support high firing rates (Perney and Kaczmarek, 1997; Golding et al., 1999).

These intrinsic and synaptic properties shape the *in vivo* responses of octopus cells. They typically respond weakly to sustained pure tones because of their broad frequency tuning and fast membrane time constant (Godfrey et al., 1975; Rhode and Smith, 1986). However, when multiple AN inputs are activated near simultaneously, such as during transient broadband sounds like clicks, they fire with exceptional temporal precision, often phase-locking with exactly one spike per stimulus up to hundreds of Hertz (McGinley and Oertel, 2006; Recio-Spinoso and Rhode, 2020; Lu et al., 2022) Beyond simple sound onsets, octopus cells respond robustly to complex spectrotemporal patterns, including frequency modulations and appear sensitive to the direction and speed of frequency sweeps—features characteristic of speech and other natural sounds (Young and Sachs, 1979; Shannon et al., 1995; Pressnitzer et al., 2011; Shamma et al., 2011; Lu et al., 2022).

In summary, octopus cells are biophysically similar to MSO principal neurons, featuring an extraordinarily low input resistance and a fast membrane time constant. Combined with their broadband AN inputs, they are ideally suited to detect monaural coincidences across a wide frequency range. Thus, octopus cells may be involved in binding co-occurring frequency information and detecting monaural spectral patterns on a sub-millisecond timescale and rapidly transmitting this information to ascending brain centers, including the VNLL.

### Globular cells of the ventral nucleus of the lateral lemniscus

Within the VNLL, a key relay station in the ascending auditory pathway, globular cells are particularly crucial for temporal processing. While perhaps less prominent than nuclei in the SOC or IC, the VNLL is essential for processing precise auditory timing and is a key structure for integrating the spectral information of sounds. Primarily receiving contralateral monaural inputs, VNLL globular cells are typically inhibitory and rely on fast and reliable signal transmission to preserve the temporal features encoded in the CN (Oertel, 1999). These cells subsequently provide broadband onset inhibition to neurons within the VNLL, the SOC, and the ipsilateral IC, which might help to reduce spectral splatter and sharpen temporal responses (Adams, 1979; Brunso-Bechtold et al., 1981; Whitley and Henkel, 1984; Nayagam et al., 2005; Spencer et al., 2015; Gómez-Martínez et al., 2023).

#### Structure and morphology

Globular cells are predominantly found in the ventral portion of the VNLL in most mammals (Adams, 1979, 1997; Covey and Casseday, 1986; Merchán et al., 1994; Schofield and Cant, 1997; Zhao and Wu, 2001; Mylius et al., 2013; Zacher and Felmy, 2024). In bats, these cells are arranged in a distinct columnar area, perhaps representing a structural adaptation linked to echolocation (Zook and Casseday, 1985; Covey and Casseday, 1986). Morphologically, globular cells feature round to oval cell bodies with extensive dendritic trees, bearing some resemblance to bushy cells in the CN and MNTB principal neurons (Adams, 1979, 1997; Covey and Casseday, 1986; Kudo et al., 1990; Vater and Feng, 1990; Merchán et al., 1994; Schofield and Cant, 1997; Vater et al., 1997; Zhao and Wu, 2001; Mylius et al., 2013). Similar to those related cell types, a defining characteristic of VNLL cells is their primary input from a giant axosomatic pre-synaptic terminal. These VNLL endbulbs are glutamatergic terminals originating from the thick axons of octopus cells located in the contralateral CN (Friauf and Ostwald, 1988; Vater and Feng, 1990; Schofield, 1995; Adams, 1997; Schofield and Cant, 1997; Vater et al., 1997; Smith et al., 2005).

Like the endbulbs in the CN and the calyx in the MNTB, VNLL endbulbs represent a conserved anatomical adaptation found in several mammals, including cats (Stotler, 1953; Adams, 1997; Smith et al., 2005), rodents (rat: Friauf and Ostwald, 1988; gerbil: Berger et al., 2014; mouse: Caspari et al., 2015), guinea pigs (Schofield and Cant, 1997), bats (Covey and Casseday, 1986; Vater and Feng, 1990; Huffman and Covey, 1995; Vater et al., 1997), and humans (Adams, 1997). Intriguingly, these large terminals are present even in primates and humans, where the VNLL is considered relatively poorly developed compared to other species. Notably, although VNLL endbulbs have been found in several mammalian species, the percentage of VNLL cells receiving endbulbs is significantly higher in humans than in cats, despite an overall less developed nucleus (Adams, 1997). Although comprehensive data on the presence and size of VNLL inputs across species are currently lacking, the high proportion of endbulb-receiving VNLL neurons in humans may indicate an adaptation to low-frequency hearing.

#### Organization of synaptic inputs

While morphologically similar to CN endbulb and calyx terminals, VNLL endbulbs are less extensively studied. Most globular cells receive input from a small number of endbulb inputs, with estimates ranging between 1–3 (Covey and Casseday, 1986; Vater and Feng, 1990; Adams, 1997; Berger et al., 2014; Baumann and Koch, 2017; Kladisios et al., 2020). In terms of convergence, this places them between bushy cells (often receiving multiple endbulbs) and MNTB cells (typically receiving a single calyx). Correspondingly, VNLL endbulbs are approximately half the size of the calyx terminal (Berger et al., 2014). Unlike the intricate branching observed at the CN endbulb and the calyx, VNLL endbulbs are morphologically simpler, with less complex branching and fewer processes, essentially clasping the globular cell soma (Adams, 1997). Ultrastructurally, VNLL endbulbs contain multiple AZs (Smith et al., 2005), rendering them a smaller and simpler version of the calyx (Berger et al., 2014).

#### Pre- and Post-synaptic physiology

Physiologically, VNLL endbulbs share key features with their CN and MNTB counterparts. Pre-synaptic APs are brief (~200 μs halfwidth), although smaller in amplitude than those in the calyx (Berger et al., 2014). However, the smaller AP is offset by changes in VGCC activation and deactivation, resulting in comparable pre-synaptic calcium influx and SV release kinetics (Berger et al., 2014).

Post-synaptically, VNLL globular cells express fast-gating AMPA receptors composed of GluA2 and GluA4 subunits (Caicedo and Eybalin, 1999) and only a minor NMDA component (Kladisios et al., 2022). This composition generates large and rapid EPSCs (<0.5 ms), similar to those in CN bushy cells and MNTB neurons (Caspari et al., 2015; Baumann and Koch, 2017; Kladisios et al., 2020). Under physiological conditions, synaptic short-term plasticity is characterized by initial facilitation followed by depression (Caspari et al., 2015; Kladisios et al., 2022), similar to the calyx of Held—MNTB synapse.

VNLL globular cells express Kv1.1 channels (Rosenberger et al., 2003; Zacher and Felmy, 2024) and moderate levels of HCN1/2 channels (Koch et al., 2004; Caspari et al., 2015; Gessele et al., 2016; Zacher and Felmy, 2024). These contribute to membrane properties closely resembling those of MNTB neurons, with an input resistance of 100–150 MΩ, a membrane time constant of approximately 5 ms, and the generation of a single or a few APs in response to depolarizing current injection (Zhao and Wu, 2001; Caspari et al., 2015; Franzen et al., 2015; Baumann and Koch, 2017; Fischer et al., 2018; Kladisios et al., 2020).

Considering the large pre-synaptic terminal together with morphological and physiological properties comparable to MNTB neurons, it is unsurprising that *in vivo* responses of VNLL globular cells to sound generally resemble those of their primary excitatory input, the octopus cells. To pure tones, these cells respond with sharply timed onset spikes independent of sound intensity and can entrain to high-frequency click trains up to 1 kHz (Covey and Casseday, 1991; Adams, 1997; Zhang and Kelly, 2006; Recio-Spinoso and Joris, 2014; Recio-Spinoso and Rhode, 2020).

In summary, VNLL globular cells exhibit morphological and physiological features similar to those of other fast-processing auditory brainstem neurons, such as bushy cells and MNTB neurons. The presence of such features outside the canonical sound localization pathway indicates that they are not unique to the pathways of binaural integration but rather represent a fundamental biological toolkit optimized to preserve or enhance temporal fidelity with sub-millisecond precision. Consequently, VNLL globular cells play a crucial role in faithfully relaying the precise temporal patterns encoded by their octopus cell inputs. This reliable relay is critical for processing sound onsets and complex temporal structures relevant for speech and other environmental sounds. Therefore, the existence of these “high-fidelity” features in a predominantly monaural pathway underscores the importance of rapid temporal processing across multiple auditory streams beyond binaural spatial hearing.

## Functional consequences in relation to synaptic specializations

To fully appreciate the morphological and physiological specializations discussed here, they must be viewed within their functional context. The widespread existence of these features across various species suggests strong evolutionary pressure favoring rapid, precise, and reliable neuronal signaling, which is largely invariant to the acoustic environment. Large axosomatic terminals, as seen in the CN, MNTB, and VNLL, provide a structural basis for delivering strong and rapid excitation suitable for high firing rates. As detailed above, this capability is underpinned by large SV pools and efficient VGCC-SV coupling. This pre-synaptic capacity is complemented post-synaptically by features such as fast-gating AMPA receptors, electrotonic compactness, and the expression of Kv1 and Kv3 potassium channels, all of which support fast post-synaptic responses.

However, how these specializations translate into the neuron's response to sound can vary significantly between synapses. The neuronal code containing acoustic information can be modified through selective AP transmission across the synaptic junction, creating vastly different input–output functions. Therefore, studying this relationship is crucial for understanding a neuron's computational role within its neuronal circuit. However, obtaining such input–output functions for single neurons is challenging, particularly *in vivo*.

Fortunately, the large axosomatic terminals in the CN, MNTB, and VNLL offer a unique opportunity to assess synaptic reliability *in vivo*, as extracellular recordings from these neurons often reveal complex waveforms, as noted in some of the earliest recordings (Pfeiffer, 1966; Guinan and Li, 1990; Adams, 1997). This composite signal arises from the proximity of the recording electrode to both the giant pre-synaptic terminal and the post-synaptic soma (Lorteije et al., 2009; Typlt et al., 2010). It typically starts with a small voltage deflection, the “pre-potential,” which reflects the pre-synaptic AP. A few hundred microseconds later, a second potential appears, generated by the EPSP, which may or may not be large enough to trigger a post-synaptic AP. When transmission fails (i.e., no post-synaptic AP is triggered), the isolated EPSP becomes clearly visible, as seen in recordings from the mature endbulb or pre-hearing calyx terminals (Lorteije et al., 2009; Typlt et al., 2010; Crins et al., 2011; Kuenzel et al., 2011; Sonntag et al., 2011; Keine and Rübsamen, 2015; Keine et al., 2016; Stasiak et al., 2018). When transmission succeeds, the EPSP typically merges into the rising phase of a much larger post-synaptic AP, often remaining as a discernible *bump*. Statistical analysis of their temporal relationships combined with pharmacological studies to isolate the origin of these components provides robust evidence for this interpretation of the complex waveform (Mc Laughlin et al., 2008; Englitz et al., 2009; Lorteije et al., 2009; Typlt et al., 2010; Sonntag et al., 2011; Keine and Rübsamen, 2015; Keine et al., 2016; Stasiak et al., 2018). These recordings thus provide a powerful tool for assessing input–output functions *in vivo* and help elucidate how AP failures affect sound encoding.

Despite sharing many structural and morphological features, the endbulb-BC and calyx-MNTB synapses display markedly different synaptic reliability and input-output functions. In mature animals, the calyx of Held-MNTB synapse is renowned for its high fidelity. MNTB neurons are among the few cells in the central nervous system in which the output is typically a one-to-one representation of its calyceal input, allowing the synapse to act as a reliable relay for auditory information. While some studies found occasional transmission failures *in vivo* (Guinan and Li, 1990; Kopp-Scheinpflug et al., 2003; Lorteije et al., 2009; Crins et al., 2011; Wang et al., 2015), others reported reliable synaptic transmission (Green and Sanes, 2005; Mc Laughlin et al., 2008; Englitz et al., 2009; Sonntag et al., 2009, 2011; Tolnai et al., 2009; Stasiak et al., 2018). In some cases, statistical analysis suggests that post-synaptic APs from another, more distant cell were mistakenly interpreted as isolated pre-potentials (Mc Laughlin et al., 2008; Englitz et al., 2009). Nonetheless, discrepancies might also arise from methodological factors, including potential cell damage from the recording electrode, differences in recording conditions, and the impact of anesthesia. Under most conditions, however, synaptic transmission at the calyx-MNTB synapse appears to be highly secure. Consequently, the *in vivo* response of MNTB neurons to sound is virtually identical to that of their corresponding GBC inputs, characterized by a primary-like response pattern with a more or less prominent “notch” in the peristimulus-time histogram (Smith et al., 1998; Rhode, 2008; Sonntag et al., 2009). Although MNTB neurons receive strong inhibitory inputs with fast kinetics, their functional impact remains unclear (Albrecht et al., 2014; Mayer et al., 2014).

This high fidelity stands in stark contrast to the endbulb of Held—BC synapses, which frequently fails to trigger post-synaptic APs. These failures, characterized by the presence of the pre-potential and an isolated EPSP but lacking the post-synaptic AP, are observed across species even during spontaneous activity and are particularly recognizable in SBCs that receive large endbulb inputs (Englitz et al., 2009; Kuenzel et al., 2011; Nerlich et al., 2014b; Keine and Rübsamen, 2015; Keine et al., 2016, 2017; Stasiak et al., 2018). This indicates that, unlike the calyx, the endbulb operates near the bushy cell's AP threshold, rendering signal transmission and, thus, the neuron's input–output function highly sensitive to modulation, especially by inhibition (Englitz et al., 2009; Dehmel et al., 2010; Kuenzel et al., 2011; Keine and Rübsamen, 2015; Keine et al., 2016, 2017; Stasiak et al., 2018). While bushy cell responses to pure tones often resemble the primary-like pattern of their AN input (Blackburn and Sachs, 1989; Rhode, 2008; Typlt et al., 2012), the interplay between strong endbulb-evoked excitation, local inhibition, and convergence of multiple inputs enhances temporal precision and aids in encoding the temporal aspects of sound (Dehmel et al., 2010; Kuenzel et al., 2011; Keine and Rübsamen, 2015; Keine et al., 2016, 2017; Koert and Kuenzel, 2021; Spirou et al., 2023).

Data on the synaptic reliability of VNLL endbulbs are limited. *In vitro* studies suggest that while a single endbulb might be insufficient to reliably trigger post-synaptic APs before hearing onset (Berger et al., 2014), a developmental increase in EPSC size (Baumann and Koch, 2017; Kladisios et al., 2020) leads to a reliable one-to-one connection in mature animals, although it may be less robust than the calyx at high stimulation frequencies (Caspari et al., 2015; Kladisios et al., 2020). Consistent with this, *in vivo* recordings in mature cats have found no evidence of isolated pre-potentials, suggesting reliable transmission (Adams, 1997). Whether the reliability of the mature VNLL endbulb is modulated during ongoing activity and whether species-dependent differences exist remains to be investigated.

These differences in reliability likely reflect the different functions within the neuronal circuit. It is conceivable that the computational roles diverge when large excitatory inputs target inhibitory vs. excitatory neurons. In sign-inverting synapses, such as those in the MNTB and VNLL, excitatory input is inverted into an inhibitory output to multiple brain areas. If the inversion of sign is the main purpose of these synapses, it is perhaps unsurprising that they exhibit high reliability with little to no modulation of the input-output function. Conversely, at the CN endbulb-bushy cell synapses, where both the auditory nerve input and bushy cell output are excitatory, the junction allows for more complex synaptic integration and signal modulation. This modulation can manifest as an increase in temporal precision, reproducibility, and sparsity of the bushy cell output compared to the auditory nerve (Dehmel et al., 2010; Kuenzel et al., 2011; Keine and Rübsamen, 2015; Keine et al., 2016, 2017).

## Comparison to other brain areas

Although the specializations that enable rapid and precise signaling in the auditory brainstem are remarkable adaptations, they are not unique to the auditory system. Similar adaptations can be observed in other brain areas facing comparable challenges. Many of these specializations are widespread across different neuronal populations and species, suggesting common solutions to universal problems in high-frequency neurotransmission.

Generally, achieving high-frequency synaptic transmission requires several key elements: fast and efficient SV release, rapid gating of post-synaptic receptors, and brief action potentials that allow the neuron to generate them in quick succession. For instance, the tight nanodomain coupling between VGCC and SV that ensures rapid nanodomain release has been observed far beyond mammalian auditory synapses, including the frog neuromuscular junction (Harlow et al., 2001; Shahrezaei et al., 2006), the squid giant synapse (Augustine, 1990; Adler et al., 1991), and the ciliary ganglion of the chick (Stanley, 1993). Efficient coupling between SV and VGCC is not limited to excitatory synapses but also found in inhibitory neurons of the cerebellum and cortex (Bucurenciu et al., 2008; Arai and Jonas, 2014). Similarly, the expression of fast calcium sensors Syt1 or Syt2 is a common feature of synapses requiring fast exocytosis, appearing in both excitatory and inhibitory synapses across various brain regions, including the brainstem, cerebellum, hippocampus, and neocortex (Geppert et al., 1994; Fernández-Chacón et al., 2001; Pang et al., 2006; Xu et al., 2007; Kochubey et al., 2016).

Post-synaptically, fast-gating AMPA receptors are crucial for generating rapid excitatory synaptic currents, minimizing spike time jitter, and maintaining high temporal precision (Cathala et al., 2005; Takahashi, 2005). Such receptors are expressed in various brain areas, including the olfactory bulb (Schoppa, 2006), the vestibular calyx synapse (Kirk et al., 2017), and fast-spiking cortical interneurons (Hull et al., 2009). Furthermore, high-threshold, voltage-gated potassium channels of the Kv3 type support the ability to fire rapidly and are found in fast-spiking neurons across different brain regions, such as the neocortex, hippocampus, subthalamus, amygdala, the vestibular nucleus, and even the electrosensory system of weakly electric fish (Weiser et al., 1995; Du et al., 1996; Massengill et al., 1997; Sekirnjak et al., 1997; Martina et al., 1998; Chow et al., 1999; Erisir et al., 1999; Wigmore and Lacey, 2000; Rashid et al., 2001; Tansey et al., 2002; McDonald and Mascagni, 2006; Gittis et al., 2010). These channels are activated only briefly near the peak of an AP, due to their high voltage threshold, causing rapid repolarization and prominent after-hyperpolarization. This shortens the AP duration and facilitates the recovery of voltage-gated sodium channels, enabling neurons to sustain high firing rates without compromising AP amplitudes.

A compelling example of specialization for high-frequency signaling occurs in the cerebellum, specifically at the synapse between cerebellar mossy fiber boutons (cMFB) and granule cells (GC). Granule cells can maintain extraordinarily high firing rates, exceeding 1000 spikes/second (Garwicz et al., 1998; Rancz et al., 2007; Prsa et al., 2009; Ritzau-Jost et al., 2014). Although cMFB are morphologically conventional bouton-type synapses, they are significantly enlarged (3–12 μm) (Hámori and Somogyi, 1983; Rothman et al., 2016), making them accessible for direct pre-synaptic patch-clamp recordings both *in vitro* and *in vivo* (Rancz et al., 2007; Powell et al., 2015). Unlike bushy cells and MNTB neurons that receive one or a few large axosomatic terminals, GCs integrate inputs from many cMFBs (12–50) (Jakab and Hámori, 1988; Billings et al., 2014). Each input contains multiple AZs, totaling 150–300 AZs per GC (Jakab and Hámori, 1988; Xu-Friedman and Regehr, 2003; Kim et al., 2013).

Mirroring the auditory synapses described earlier, SV release in the cMFB is triggered by extremely brief pre-synaptic APs (Ritzau-Jost et al., 2014). Pre-synaptic calcium entry is predominantly mediated by Ca_V_2.1 channels tightly coupled to SVs, leading to fast nanodomain release, similar to many synapses in the auditory brainstem (Sargent, 2005; Delvendahl et al., 2015a). Reliable high-frequency transmission is sustained by a large RRP and efficient SV replenishment (Saviane and Silver, 2006; Hallermann et al., 2010; Ritzau-Jost et al., 2014). Furthermore, pre-synaptic calcium-buffer dynamics are similar to those observed in the calyx of Held (Helmchen et al., 1997; Jackson and Redman, 2003; Müller et al., 2007).

Post-synaptically, GCs are smaller than bushy cells or MNTB cells (5–7 μm in diameter) but display a similar electrotonic compactness due to their short dendrites, which minimize dendritic filtering (Silver et al., 1992; D'Angelo et al., 1993; Delvendahl et al., 2015b), and thus resemble the adaptations observed in bushy cells, MNTB cells, and MSO principal cells.

Similar to auditory brainstem neurons, GCs use fast-gating AMPA receptors composed of GluA2 and GluA4 subunits, which enable rapid synaptic currents and efficient recovery from desensitization (Silver et al., 1992; Cathala et al., 2005; DiGregorio et al., 2007). The ability to fire rapidly is also supported by the expression of Kv1 and Kv3-type potassium channels (Ritzau-Jost et al., 2014). Notably, unlike in most mature auditory brainstem neurons, where NMDA receptor expression is low, these receptors are substantially expressed in GCs (D'Angelo et al., 1990; Silver et al., 1992). Additionally, GCs exhibit a slow component of their AMPA receptor currents, which may contribute to sustaining elevated firing rates by producing tonic depolarization (Digregorio et al., 2002; Xu-Friedman and Regehr, 2003; Nielsen et al., 2004; Sargent, 2005; Rancz et al., 2007; Powell et al., 2015).

In summary, the specializations observed in the auditory brainstem are not isolated phenomena. Similar structural and physiological adaptations appear in other brain regions that face comparable computational demands. This suggests that mechanisms such as nanodomain coupling, specific calcium sensors and specialized receptors and ion channels represent a fundamental molecular toolkit that evolution has employed and refined repeatedly to meet the diverse functional demands of neural circuits throughout the brain.

## Discussion

A core principle in neuroscience is that distinct neural circuits have evolved with specialized properties to perform specific computations (Luo, 2021). The auditory system is inherently temporal, requiring the extraction of relevant features from rapidly varying sound pressure waves; thus, it faces unique challenges compared with other sensory systems. Consequently, the importance of the temporal dimension has resulted in several structural and functional specializations along the auditory pathway. While the cochlea encodes basic features such as sound intensity and frequency, more complex features such as sound localization, source segregation, and auditory object identity emerge from computations performed along the auditory pathway, starting in the auditory brainstem. The fundamental computations (localization, timing, and spectral analysis) performed at the auditory brainstem then act as building blocks for further processing in higher auditory centers to achieve complex perception.

Perhaps the most widely recognized function requiring exquisite temporal precision is the localization of sound sources in the horizontal plane based on ITDs and ILDs. Human listeners can detect ITDs as small as 10–20 μs (Klumpp and Eady, 1956; Mills, 1958), requiring neuronal computations to operate at sub-millisecond precision. Beyond spatial hearing, understanding complex sounds, such as speech, relies on this temporal acuity to encode rapid transients (e.g., consonant bursts), periodic fine structure (pitch), and slower envelope modulations (syllables) (Russo et al., 2004; Akhoun et al., 2008). Octopus cells in the PVCN, with their rapid membrane properties and broadband AN inputs, are ideally suited to detect the coincident firing of many AN with exceptional temporal precision. This precise temporal information is then relayed via the VNLL globular cells, which provide fast and reliable inhibition, perhaps crucial for segmenting speech streams, identifying consonants, and reducing spectral splatter (Nayagam et al., 2005; Spencer et al., 2015). Furthermore, the ability to encode temporal fine structure is vital for pitch perception and contributes significantly to vowel identification and understanding speech in noisy or reverberant environments (Lorenzi et al., 2006; Moore, 2008; Hopkins and Moore, 2011). Ultimately, auditory scene analysis, the ability to segregate sound sources in the environment (“cocktail-party” problem), depends on the high-resolution spatial and temporal information encoded by the brainstem (Shamma et al., 2011; Yao et al., 2015).

The perception of acoustic stimuli is modulated by the temporal and spectral relationships between sound components, and these interactions can be used to segregate sound sources, such as multiple speakers in a room (Palmer, 1990; de Cheveigné, 2021). The octopus cell—VNLL pathway appears to be particularly sensitive to such interactions (Recio-Spinoso and Rhode, 2020; Lu et al., 2022). This manifold use of temporally precise auditory information underscores the critical importance of the brainstem's specialized machinery in preserving the fine temporal details necessary for hearing in naturalistic settings.

In conclusion, the specialized cellular and synaptic properties found within auditory brainstem pathways, including giant synapses, cellular morphology, and ion channel expression patterns, constitute a highly optimized biological framework designed for computations that demand extraordinary temporal precision. Understanding the molecular and cellular basis of this temporal precision is crucial for deciphering the neural basis of hearing in both normal and pathological states. Future studies that use cell-type-specific optogenetics (Mattis et al., 2011), transcriptomics (Jing et al., 2025), and population recording techniques, such as high-density electrode arrays (Steinmetz et al., 2021), will be essential for advancing our understanding of the circuits formed by neurons and synapses discussed in this review.
